# Assessing Questionable and Responsible Research Practices in Psychology Master’s Theses

**DOI:** 10.3390/bs16010110

**Published:** 2026-01-13

**Authors:** Hilde E. M. Augusteijn, Jelte M. Wicherts, Klaas Sijtsma, Marcel A. L. M. van Assen

**Affiliations:** 1 Department Methodology and Statistics, Tilburg University, 5037 AB Tilburg, The Netherlands; h.e.m.augusteijn@tilburguniversity.edu (H.E.M.A.); j.m.wicherts@uvt.nl (J.M.W.); k.sijtsma@uvt.nl (K.S.); 2 Department of Sociology, Utrecht University, 3584 CS Utrecht, The Netherlands

**Keywords:** responsible research practices, questionable research practices, education, master’s thesis, statistical reporting

## Abstract

Recent research has documented both questionable and responsible research practices in published psychology research, but it is unclear which research practices psychology students engage in when graduating from their master’s program. In this study, we documented the prevalence of responsible and questionable research practices in 300 psychology master’s theses from Tilburg University, the Netherlands, and associated these practices with supervisor’s grading of the theses. Compared to authors of published scientific manuscripts, master’s students seemed to engage more in responsible research practices, conducted more power analyses, used larger sample sizes, reported fewer statistically significant results, and provided more detailed reporting of their results. However, statistical reporting errors were almost as common in master’s theses as they are in the published literature. We found no relationship between thesis grades and any of the responsible or questionable research practices. We also found no relationships among practices, suggesting that there is no unidimensional construct of “responsible scientific behavior”.

## 1. Introduction

It has been claimed that most published research findings are false (Ioannidis, 2005). The low replicability rate in psychology supports this claim (Klein et al., 2018; Open Science Collaboration, 2015). The combination of low statistical power (Bakker et al., 2012; Cohen, 1990) and the large prevalence of statistically significant results in the published psychology literature (Fanelli, 2010) suggests that common suboptimal research practices create an overrepresentation of statistically significant findings in the literature. Researchers may be less inclined to submit their manuscripts with a statistically non-significant main finding (known as the file drawer problem), journals may prefer manuscripts with significant main findings (known as publication bias), and researchers may engage in questionable research practices (QRPs) to increase the probability of obtaining a statistically significant finding (also known as *p*-hacking).

QRPs in the analysis and reporting of outcomes result in the increase in type I errors, an increase in statistical power if the null-hypothesis is false (Manapat et al., 2022; Simmons et al., 2011), inflations in estimated (true) effects (Bakker et al., 2012; Moran et al., 2023), and subsequent failures to replicate. Examples of QRPs are selective reporting of (dependent) variables, conducting multiple analyses on a data set until the desired result is achieved, selective reporting of results that support the hypothesis, and hypothesizing after the results are known while presenting the hypothesis as confirmatory (also referred to as HARKing, (Kerr, 1998)). Most QRPs are not the result of bad intentions (Sijtsma, 2016) but can also result from lack of statistical knowledge and human biases, such as confirmation bias or hindsight bias (Veldkamp et al., 2017). QRPs are a threat to the validity of research claims and degrade the quality of scientific work.

Many initiatives have been launched to counter QRPs and to improve the quality and transparency of research, such as preregistration and data sharing (Kidwell et al., 2016; Miguel et al., 2014). Awareness of the importance of ethical research behavior is widespread (e.g., Bravin et al., 2020; Godecharle et al., 2014; Hofmann et al., 2013; Kandeel et al., 2011; Löfström, 2012; Okoroafor et al., 2016), and there are many initiatives to help researchers engage in responsible research practices (RRPs). For example, more and more journals stimulate open science practices, preregistration, and registered reports (Hardwicke et al., 2024; Reich, 2021). Another initiative is to improve the education of future researchers. However, our own human biases (Kahneman, 2011; Kahneman et al., 1982), and all the published literature our students read during their education, may impact our students in a negative way. Furthermore, the perceived attitude of the teacher toward QRPs impacts the students’ attitude and their behavior towards these QRPs (Krishna & Peter, 2018) and sets the bar for what is considered high-quality research. The type of mentoring students receive influences students’ probability of engaging in QRPs. Mentoring focused on survival in the research field has shown to be associated with higher use of QRPs by the student (Anderson et al., 2007), whereas responsible mentoring is associated with lower use of QRPs and higher use of RRPs (Gopalakrishna et al., 2022a, 2022b).

At the end of their master’s program, students write a research thesis, which is their final aptitude test that should reflect what they have learned and internalized during their education. In their thesis, students apply their acquired skills and have the chance to employ both RRPs and QRPs (Krishna & Peter, 2018). In this research project, we coded characteristics of many psychology master’s theses that indicate RRPs and QRPs and investigated how these characteristics are related to the grade students received for their thesis. This allowed us to answer the main research question of this study:

What evidence of RRPs or QRPs do psychology master’s theses show, and what do their supervisors reward when they grade this final exam?

Next, we will discuss what characteristics of a thesis are evidence of RRPs or QRPs and why we classify them as such.

### 1.1. Responsible Research Practices (RRPs)

In manuscripts reporting quantitative hypothesis testing, many characteristics like preregistration, power analysis, large sample sizes, and thorough reporting of conducted analyses and results indicate RRPs. We discuss what is currently known about the use and prevalence of RRPs in academic literature and in students’ theses.

First, several researchers consider *preregistration* a possible solution to the replicability crisis, since it limits the opportunistic use of researchers’ degrees of freedom (i.e., the many choices researchers make during a study, such as which variables to analyze, which participants to exclude, or when to stop data collection (Simmons et al., 2011)) and *p*-hacking (Bakker et al., 2020). If a preregistration is sufficiently detailed, it provides insight on what was planned prior to the data collection and, therefore, which decisions, hypotheses, and analyses were confirmatory and which choices were post hoc. Preregistration has become increasingly popular in the social sciences. For example, the number of preregistrations at OSF approximately doubles every year (Bakker et al., 2020), with over 550,000 registries mid-2022 (see http://osf.io/registries). Despite this growth in absolute numbers, a random sample of 250 psychology articles published between 2014 and 2017 showed that only 3% of these papers were preregistered (Hardwicke et al., 2021), and only about 7% in the field of psychology in 2022 (Hardwicke et al., 2024). Even though many, or perhaps most, student thesis projects do not aim for a publication, preregistration of the research proposal at, for instance, AsPredicted is a relatively simple procedure, especially since students often write down their research plan for their supervisors, including many of the details that would also be included in the preregistration (Pownall, 2020). Although we are not aware of any data on the prevalence of preregistration of student theses, at some universities, preregistration is already part of the psychology curriculum (Blincoe & Buchert, 2020). Furthermore, Pownall et al. (2023) investigated the pedagogical effectiveness of preregistering the undergraduate thesis.

With or without preregistration, any research report can have characteristics of RRPs. A first example of RRPs in the context of preregistration is *explicit listing of the hypotheses*, which should force the writer to formulate precise hypotheses. Second, providing a *clear distinction between confirmatory and exploratory hypotheses and analyses* (Pownall, 2020; Wagenmakers et al., 2011) helps the reader to evaluate the evidence the manuscript provides. Confirmatory hypotheses should be evaluated differently than tests conducted without prior expectations. Often, researchers do not provide such a distinction. For example, covariates are included, tests are conducted on multiple sub-scales, or outliers are removed, all without providing motivation (Wicherts et al., 2016).

Another RRP is having a sufficiently *large sample size* in a study and hence achieving sufficient power (if the null hypothesis H0 is incorrect). Effect sizes within psychology are relatively small, usually between small and medium (Gignac & Szodorai, 2016; Hartgerink et al., 2017; Nuijten et al., 2020; Open Science Collaboration, 2015; Szucs & Ioannidis, 2017). In a typical two-group between-subjects design, with significance level α = .05 and effect size *d* = 0.35, an independent samples *t*-test requires a sample size of 204 to achieve a power of 80%. However, the median sample size reported in published articles in psychology is only 62 (Hartgerink et al., 2017), which corresponds to a median power of .39, using the same independent *t*-test, suggesting that many studies would have been severely underpowered were they using a between-subjects design (Bakker et al., 2012; Szucs & Ioannidis, 2017). Due to the limited time available for a master’s thesis, students are sometimes provided with a data set by their supervisor or data are collected in collaboration with other students to increase the sample size. A study on 250 economic psychology master’s theses at the University of Vienna showed a median sample size of 157, and a median power of .67 (based on reported effect sizes), much larger than the median sample size of 62 in the published literature (Olsen et al., 2019).

Reporting an appropriate *power analysis* is another RRP. A power analysis can be conducted prior to the data collection to determine the minimum number of participants needed to achieve sufficient power to detect an expected effect size. If the sample size is predetermined, power analysis can be used to determine the minimum effect size one could find with sufficient power (e.g., 80%). A power analysis can also be conducted after data have been collected, for example, to test if the sample size is sufficient for the planned analysis or as part of an attempt to explain absence of significant results. An a priori power analysis for sample size planning is preferred, but this is not always feasible, for example, because the sample size is limited due to practical or financial constraints. Olsen et al. (2019) showed that only one thesis in their sample reported an a priori power analysis, whereas approximately one third of the students in the study by Krishna and Peter (2018) reported that they conducted a power analysis. Note, however, that the research cited here and ours did not check whether the reported power analysis was appropriate for the research.

Another RRP is the *thorough reporting of statistics*, such as effect sizes, confidence intervals, and checking the underlying assumptions of statistical tests. This allows readers to better evaluate the effect and its strength, and how these were obtained. Approximately 38% of published psychology articles provided effect sizes, and approximately 10% provided confidence intervals (Fritz et al., 2013). Unfortunately, many statistics such as validity and reliability statistics were often poorly reported (Barry et al., 2014; Flake & Fried, 2020; Oosterwijk et al., 2019). Krishna and Peter (2018) found that 69% of the students claimed they reported effect sizes in their final theses. The lack of reported statistical details impedes verification of the statistical results, enables QRPs, and complicates replication studies and meta-analysis (Fanelli, 2013; Flake & Fried, 2020).

Finally, *transparent sharing and reporting practices* is also an RRP. Examples are sharing research materials, data, and analysis scripts. Over 100 journals (https://www.cos.io/initiatives/badges, assessed on 7 January 2026) currently use open science badges for data and material sharing. These badges seem to be a success incentive of providing open data and materials (Kidwell et al., 2016). Transparent sharing and reporting practices are still rare. Hardwicke et al. (2021) found that materials were shared in 14% of the published papers, raw data in 2%, and only 1% of the articles shared analysis scripts.

### 1.2. Questionable Research Practices (QRPs)

QRPs are practices related to study design, data analyses, or reporting that can artificially increase the likelihood of finding evidence in support of a specific hypothesis or present the evidence in a biased way to achieve a particular conclusion (John et al., 2012). John et al. (2012) found that over 90% of psychology researchers have engaged in a QRP at least once in their academic career. This is in line with Makel et al. (2021), who found that only 8.6% of participants claimed they had never engaged in a QRP. Furthermore, 47% of Italian psychology researchers indicated that they were tempted to adopt one or more QRPs at least once in the past year (Agnoli et al., 2017), 18% of American psychologists admitted that they had engaged in QRPs in the past year (Fox et al., 2022), and 51.3% of Dutch researchers indicated they frequently engaged in at least one QRP (Gopalakrishna et al., 2022a). Students are also no stranger to QRPs, as Krishna and Peter (2018) found that more than 40% of the psychology students in their study had engaged in at least one QRP in their thesis, and Moran et al. (2023) found that 64% (up to graduate level) had already engaged in at least one QRP during their short academic careers.

QRPs are different from research misconduct, summarized as Fabrication, Falsification, or Plagiarism (FFP, or research misconduct), which is usually intentional (Dal-Ré et al., 2020). Luckily, research misconduct is much scarcer than QRPs; estimates of research misconduct range between 2% (Fanelli, 2009) and 8% (Gopalakrishna et al., 2022a). Engaging in a QRP once is usually not a major violation of research ethics and most researchers try to conduct science in the best way possible. QRPs are often not the result of bad intent, but rather the result of research habits, unawareness of its problematic consequences, and human biases. However, QRPs are problematic, since they result in false positives, unreplicable results, wasted time and money, and erosion of public trust in science.

An example of a QRP is inaccurate reporting of statistical results. *Statistical reporting errors* are common in the literature. A large study considering over 16,000 psychology articles containing null hypothesis significance testing results showed that almost 50% contained at least one inconsistency, and 12.9% contained a gross inconsistency that would change the conclusion of the results (Nuijten et al., 2016). We would hope that both researchers and peer reviewers read, re-read, and check their results, and that inaccurate reporting does not occur as often as it does. The same holds for authors of master’s theses. Given that it is their final report, and knowing it will be graded and scrutinized, students should check and recheck their results and their reporting. Olsen et al. (2019) showed that in economic psychology master’s theses, 18% of the focal hypothesis tests contained an inconsistency, whereas 2% contained a gross inconsistency in the reporting of the focal hypothesis results.

Another QRP is *conducting multiple statistical tests of the same hypothesis*. Researchers can analyze their data in many ways and with many different tests (Gelman & Loken, 2013). For example, data can be analyzed with and without outliers, items of a scale can be dropped, and analysis can be performed using multiple dependent variables. When a researcher conducts multiple statistical tests but does not report all of them, does not adjust for multiple testing (e.g., with a Bonferroni correction), or decides whether the null hypothesis should be rejected on an arbitrary (sub)set of these results, this is a QRP. This opportunistic use of researchers’ degrees of freedom increases the probability of type I errors. When reading a scientific manuscript, it is usually not possible to know which statistical tests were conducted, since the reader only sees the results that were reported. Information about testing is transparent and trustworthy when an analysis plan was preregistered, and the reported results concerning the planned hypotheses exactly match the preregistration or emphasize any differences.

Many QRPs and researchers’ degrees of freedom can be used opportunistically (John et al., 2012; Wicherts et al., 2016). They are related to different aspects of empirical research, such as hypothesizing (e.g., studying a vague hypothesis that fails to specify the direction of the effect), research design (e.g., enabling many different analyses by creating multiple variables or conditions, measuring a variable in several different ways, or measuring additional constructs), data collection (e.g., deciding whether to collect more data after testing, whether results are significant, or stopping data collection earlier than planned because a result has been found), data analyses (e.g., analyzing results using multiple independent variables, constructs, or dependent variables to see what “works”), or reporting (e.g., selectively reporting those studies and/or experimental conditions that worked or reporting unexpected findings as if they were predicted from the start).

Some QRPs are impossible to detect based on the final manuscript alone. Furthermore, not all QRPs are equally likely in a master’s thesis. That is, conducting multiple studies requires a large time investment, and not reporting these additional studies might be less beneficial for a student than reporting only “successful” conditions or studies. In the current project, we consider evidence of QRPs in students’ master’s theses by studying statistical reporting errors resulting from inaccurate reporting or rounding off a *p*-value, the number of statistical tests of the primary hypothesis reported, and by comparing the observed with the expected number of statistically significant results given the average power of the theses.

### 1.3. Means, Motives, and Opportunity

Researchers have means, motives, and opportunities to engage in QRPs (O’Boyle et al., 2017), and it is unknown how students differ from researchers in these respects. One exception is that students are less likely to engage in study design QRPs (Krishna & Peter, 2018). Students are not always responsible for their own study design, since they do not always collect their own data, or are already provided with analysis plans.

Students are generally motivated to achieve high grades for their final thesis (Krishna & Peter, 2018). Each educational program will have its own grading criteria. Regardless of the specific weights and framing of the different aspects that will be evaluated, ideally, RRPs should be rewarded, whereas QRPs should be discouraged. Therefore, students might be more motivated to engage in RRPs than in QRPs if they believe that RRPs are rewarded with higher grades and that statistically significant results are not important to achieve a high grade. Previous research showed that students did not believe that there was a causal relation between good science and statistically significant results, or that supervisors rewarded significant results (Krishna & Peter, 2018), which means that students do not believe they need QRPs to obtain a higher grade. However, when the supervisor intends on publication of the thesis (data), interest and quality of supervision could decrease when findings are not deemed publishable due to lack of significance, and students report feelings of inadequacy (Roberts & Seaman, 2018).

Finally, students may have fewer opportunities to engage in QRPs, since senior faculty members closely supervise the thesis, and QRPs should be less likely to go undetected (Krishna & Peter, 2018; O’Boyle et al., 2017). The perceived supervisor’s attitude regarding QRPs and behavior may have a direct effect on the students’ QRP use, as well as an indirect effect via the students’ own attitudes towards QRPs (Krishna & Peter, 2018).

O’Boyle et al. (2017) compared dissertations with resulting journal publications and concluded that many articles changed, dropped, or reversed the direction of the initial hypotheses or altered data before publication. As a result, the ratio of supported to unsupported hypotheses more than doubled. This suggests that many QRPs occur between finishing the dissertation and publication of the results. Krishna and Peter (2018) investigated the prevalence of QRPs among psychology students and found that their results “do not support the conclusion that students are employing fewer QRPs than their seniors” (Krishna & Peter, 2018, p. 9). These studies suggest students’ QRP use when writing a thesis, but even more distortion when publishing.

In the current study, we studied evidence of both RRPs and QRPs in psychology master’s theses and evaluated which characteristics predict supervisors’ final grade given. The research is descriptive and exploratory. That is, we formulated no specific hypotheses prior to data collection and analysis.

## 2. Method

### 2.1. Sample

**Figure 1:**
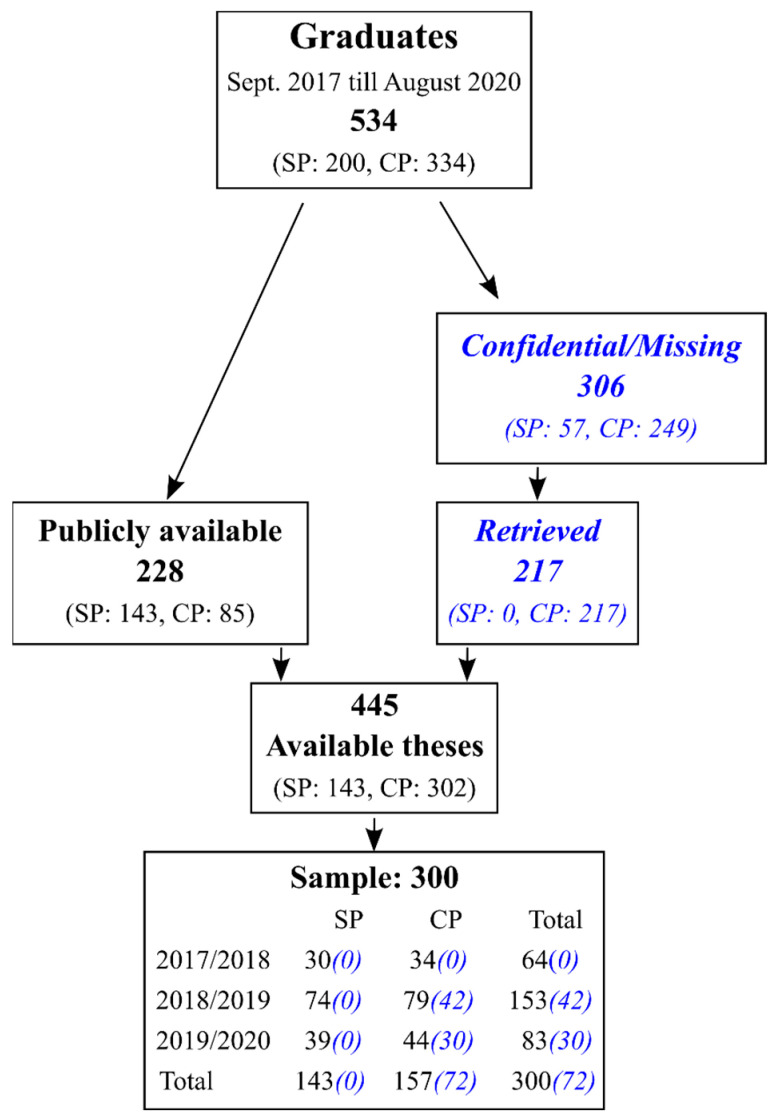
Overview of included psychology master’s theses. SP is social psychology; CP is clinical psychology. The blue text in italics indicates theses treated as confidential.

All included theses were written as part of a master’s program in psychology at Tilburg University. From the different master’s programs, all five regular one-year master’s programs were included: from the clinical psychology department, (i) Clinical Psychology, (ii) Clinical Forensic Psychology, and (iii) Clinical Child and Adolescent Psychology, and from the social psychology department, (iv) Economic Psychology and (v) Work and Organizational Psychology. The sampled theses were written in academic years 2017/2018 through 2019/2020 and were finalized between 1 September 2017 and 31 August 2020. Figure 1 provides an overview of the number of graduates and the number of available and included theses. A total of 228 theses (42.7%) were publicly available (143 from social psychology and 85 from clinical psychology), whereas 306 (57.3%) were either labeled confidential (due to sensitive data such as diagnostic data from mental health care patients, or upon request of the institute where data collection occurred) or were considered missing (not available in the library, confidentiality status unclear). An additional set of theses was retrieved from the clinical psychology department’s administration to increase the number of available theses of their study programs (programs i, ii, and iii). The confidentiality status of these theses could not be verified; to ensure that confidentiality was not breached, we treated all of these theses as confidential. A total of 32 clinical psychology theses remained missing, since they could not easily be retrieved, and no further attempt was made. Only the primary author (HA) gained access to these confidential theses and received permission to analyze them. The coded data retrieved from these confidential theses did not need to be treated as confidential.

Due to budgeting and feasibility, the intended sample size of included theses was set at 300. All publicly available theses (228) were included, and an additional set of confidential clinical psychology theses (72) was randomly sampled, while aiming for a uniform distribution over the three master’s programs. The theses were written by students who graduated their master’s program, and therefore, theses were graded as sufficient (in the Dutch grading system, this means at least 6 credit points out of 10).

### 2.2. Development of Coding Manual

A coding manual was developed by the authors H.E.M.A. and M.A.L.M.v.A. and two research assistants. H.E.M.A. and M.A.L.M.v.A. generated a list of variables that could reflect RRPs or QRPs, as well as those aspects thought to be relevant for the thesis grade. This list was the result of both brainstorming and earlier research of, for example, Olsen et al. (2019). All coded variables (see Table 1 for an overview) are discussed in the next paragraph.

Authors H.E.M.A. and M.A.L.M.v.A. developed a draft of the coding manual. Two research assistants and H.E.M.A. pretested this coding manual on a test set of five theses. After pretesting and discussion between H.E.M.A. and M.A.L.M.v.A., some variables were removed and others added, and coding instructions were changed. The coding manual was further fine-tuned after pretesting it on three additional theses by all three coders (round two). In the third round, each RA coded ten additional theses, which were double-coded by H.E.M.A. (20 in total). In round four, each RA coded an additional 10 theses (20 in total), and H.E.M.A. double-coded only those thesis characteristics that showed the most coding difficulties in the previous round (i.e., focal hypothesis and its results). One RA had remaining questions and some difficulties, and therefore, a fifth round of double-coding of the difficult items was initiated for this RA (10 theses). This time, there were no major differences in coding. Both research assistants and H.E.M.A. continued coding using the final coding protocol. Of the 58 theses used in the five protocol fine-tuning rounds, 57 were coded again using the final protocol. One thesis from the first test set was not included in the final data set, since this thesis was written before September 2017.

The theses were coded without access to the student grades to avoid undesirable influence on the coding. Grades and thesis coding were matched after all data collection was completed.

### 2.3. Coding Manual

The full coding protocol can be found in the Supplementary Materials on the OSF page of this study (https://osf.io/b4g32/). First, we coded general characteristics, such as the *cohort year* (based on the academic year 2017/2018/2019), the *master’s title*, and the *language* of the thesis. For the three clinical master’s programs, students had the option to write their thesis in either Dutch or English, whereas students of two social psychology programs were obliged to write their thesis in English. We uploaded the English student guides for the master’s programs to the OSF page. The next variable was the *thesis title*. The *length of the main text* was calculated by subtracting the page number of the abstract from the page number where the main text ends (usually after either conclusion or discussion), plus one. The *length of references and supplementary materials* was coded by subtracting the page number where the main text ended from the page number of the final page that contained any content. The *use of significance testing in the thesis* was coded as ‘Yes’ if any of the key words (significant, *p*<, *p* <, *p*=, *p* =, *p*>, *p* >, n.s.) were used, and as ‘No’ otherwise. The *number of distinct studies* within the thesis was determined by the number of new data collections, Not analyzed in other parts of the thesis, which was identified by looking at the (sub)heading. *Preregistration* of the study was checked by looking for the keywords ‘registration, research plan, register, report’, and coders checked whether a URL to the relevant preregistration was available (No/Yes, but no link/Yes, link available).

Next, we coded a set of items related to the design of the research project. First, we coded whether *hypotheses were listed explicitly* (Yes/No). The following key words were used to identify a description of the different hypotheses: ‘hypothes’, ‘predict’, ‘expect’, examin’. A listing could be provided by use of numbers, letters, bullets, in-text counting, or use of typographical emphasis (bold, underlined, italics). If an explicit listing was present, coders were asked to count the *number of explicitly listed hypotheses*, including sub-hypotheses (e.g., 2a and 2b). The listing of the author was used, except that just one hypothesis was counted in case both a null hypothesis and a matching alternative hypothesis were listed. *Moderations* or *mediations* tested were verified by searching for the key words ‘moderate, interact’ and ‘mediate’, respectively, and checking whether a moderator analysis or a mediation analysis was indeed conducted.

To determine the *sample size*, we looked at the number of participants included in the analysis of the study. If data from multiple groups were used (e.g., experimental and control condition), or if multiple studies were conducted, the numbers of participants were added. If participants were measured multiple times (e.g., repeated measures), they were counted once. The final item pertaining to the design was whether a *power analysis* was conducted. If this was the case, we determined whether the power analysis was conducted a priori (for sample size planning or determination of the maximum effect size, before data collection), post hoc (after data collection took place), or both a priori and post hoc.

Next, coders identified a *focal hypothesis*, that is, the main hypothesis investigated in the thesis. This was a relatively detailed procedure to maximize the likelihood that the same hypothesis would be coded by different coders. First, coders determined whether the title of the thesis referred to one or more of the hypotheses. If there was only one hypothesis that matched all aspects of the relationship described in the title, then this hypothesis was coded as the focal hypothesis. If multiple hypotheses matched the title, all matching hypotheses were candidate focal hypotheses; if no hypotheses matched the title, all hypotheses remained candidate focal hypotheses. Next, coders checked whether the thesis text prioritized one candidate focal hypothesis over the others by looking at whether one hypothesis was described using the words ‘key’, ‘leading’, ‘main’, ‘major’, ‘primary’, ‘principal’, or ‘important’. If this was the case, the first candidate focal hypothesis described using one of these words was coded as the focal hypothesis. If these terms were not used to prioritize one hypothesis over the others, coders would read the abstract. Then, the first hypothesis or research question mentioned in the abstract that could be linked to one of the candidate focal hypotheses was coded as focal hypothesis. If no candidate focal hypothesis was mentioned in the abstract, the candidate focal hypothesis that was listed first in the main text was coded as focal hypothesis. It was also coded in which *step the focal hypothesis was identified* by the coders.

After the focal hypothesis was identified, we coded whether the focal hypothesis described a *moderation* effect or *mediation* effect. The *number of results on the focal hypothesis* was coded by counting all results that tested the hypothesized relation. Even if results were reported as, for example, a sensitivity analysis, an additional exploratory analysis, or when the main effect of the focal hypothesis was tested again as part of another analysis, they were counted as results of the focal hypothesis. Both frequentist results and Bayesian results were counted. If there were no results concerning the focal hypothesis in the Results section(s), coders examined whether results were mentioned in the abstract. The *number of focal results that were statistically significant* was determined by counting the *p*-values smaller than .05, confidence intervals (of any width) that did not include 0, and Bayes factors larger than 3. Only these results were deemed statistically significant, regardless of interpretations of the author. *Effect size* was coded as being present if any of the results of the focal hypothesis were accompanied by an effect size (e.g., correlation coefficient, Cohens *d*, Hedges *g*, *R*^2^, η^2^, Cohens *f*^2^, ω^2^, OR, or RR), and absent if no effect size was provided.

Coders also coded what *decision* the author of a thesis made concerning the focal hypothesis. They first checked whether a decision was reported in the Abstract of the thesis. If not, they looked for a decision about the focal hypothesis in the Conclusion and/or Discussion section. When still no decision was found, coders looked for a decision in the two sentences preceding and following the results of the focal hypothesis in the Results section. Coders coded the decision as ‘Reject H_0_ (effect)’ if authors either concluded that there was an effect, concluded that they rejected the null hypothesis, or if they stated that they ‘accepted’ the alternative hypothesis. Coders coded the decision as ‘Partially reject H_0_ (some effect)’ if authors concluded that there was an effect for part of the hypothesis, concluded that they rejected part of the null hypothesis, or if they stated that they ‘accepted’ part of the alternative hypothesis. If authors concluded that there was no effect, concluded that they did not reject the null hypothesis, or they ‘rejected’ the alternative hypothesis, coders coded this as ‘Don’t reject H_0_ (no effect)’. Coders coded the decision as ‘Marginal effect’ in the case where authors concluded that there was a marginally significant effect or almost an effect or gave a reason for why the effect probably existed, even though the results were not statistically significant. If authors concluded that they found a statistically significant effect, but in the opposite direction of the original hypothesis, this was coded as ‘Effect in opposite direction’. Finally, if no decision had been provided by the authors, this was coded as ‘Inconclusive (no decision)’.

Finally, coders coded three variables related to reporting details. For *distinction between exploratory and confirmatory hypotheses and/or analyses*, they coded whether the keywords ‘confirmat’, ‘planned’, ‘explorat’, ‘additional’, ‘follow-up’ had been used in the context of stating or testing hypotheses. *Assumption tests*, tests to make sure that assumptions of statistical tests were met (e.g., normally distributed data or homogeneity of variance), were coded as present in case they were provided or in case an explanation for not conducting assumption tests was provided (e.g., due to robustness). Finally, coders coded whether confidence intervals were provided for any of the results in the thesis.

### 2.4. Statcheck

Statcheck (1.3.0, Epskamp & Nuijten, 2018) was used to analyze statistical reporting errors. The university requires that psychology theses report results in the APA format. Reported results were expected to stem from two-tailed tests. *P*-values corresponding to one-tailed testing were considered an error, unless statcheck detected a mention of one-tailed tests in the text. We did not count *p*-values reported as “*p* = 0.000” as errors, although they should have been reported as “*p* < 0.001” according to APA guidelines. The number of *p*-values per thesis detected by statcheck was added to the data set, as well as the number of inconsistencies and gross inconsistencies, that is, if the error resulted a different conclusion regarding the significance (e.g., *p* = 0.051 versus *p* < 0.05).

### 2.5. Inclusion/Exclusion Criteria

Theses that did not include null hypothesis significance tests were not coded and analyzed further. For example, this could happen when a thesis only contained a literature review, or data was only analyzed using Bayesian statistics. Although some theses did indeed contain Bayesian statistics, all 300 coded theses contained frequentist statistics. Therefore, no theses were excluded.

### 2.6. Intercoder Reliability

Seventy theses were coded by two of the coders, in changing pairs, to determine intercoder reliability. That is, the final 10 theses from the testing rounds of each SA were used; then, items difficult to code were double-coded by HA (HA-SA1: 10, HA-SA2: 10). In an additional set of 50 theses, all items were double-coded in pairs (HA-SA1: 10, HA-SA2: 10, SA1-SA2: 30). Intercoder reliabilities were estimated using Krippendorff’s alpha. Krippendorff’s alpha is an alternative to Cohen’s kappa, which can handle both missing data and varying numbers of raters per item. It can be calculated on both a nominal and ratio level, the two levels of our data. An alpha value of 0 indicates perfect disagreement, whereas an alpha value of 1 indicates perfect agreement. Krippendorff’s alpha is considered good when α ≥ 0.8, and acceptable when α ≥ 0.667 (Krippendorff, 2004).

Krippendorff’s alpha was calculated for items 3 to 26, except for items 4 and 17. Krippendorff’s alpha ranged from 0.84 to 1, except for items 20 and 22. The coders agreed only 45.8% of the time on the number of results related to the focal hypothesis (item 20, Krippendorff’s alpha = 0.68), and they agreed in only 83% of the theses (Krippendorff’s alpha = 0.66) on whether or not an effect size was reported (item 22). The latter seemed mainly due to a difference in interpretation of whether reporting a correlation was sufficient to code as an effect. For both items, coders needed to rely heavily on their own interpretation, thus introducing subjectivity.

The most important variable for coding was identification of the focal hypothesis (item 16), since many other items depended on this item. The same hypothesis was coded as the focal hypothesis in 84% of the theses (59/70). Furthermore, if coders agreed on the focal hypothesis, they also agreed in 91.5% of the theses (54/59) in their coding of the decision whether or not the null hypothesis was rejected (item 22, Krippendorff’s alpha = 0.87). For more details and intercoder reliabilities for all items, see the Supplementary Materials (https://osf.io/b4g32/).

### 2.7. Ethical Review and Data Management

This research project received an exemption for ethical review by the ethical review board of the Tilburg School of Social and Behavioral Sciences (ERB, EC-2019.EX136) because only existing or secondary data were analyzed. The data management plan was approved and the use of personal data was authorized for this research project. The data management plan described data storage, open data plans, a pre-DPIA (Data Protection Impact Assessment), and a description of GDPR agreements and compliance. The data files, not including the master’s thesis titles, and analysis code files are available on OSF (https://osf.io/b4g32).

## 3. Results

We analyzed the data using R version 4.4.3. Except for students’ grades, anonymized data, R-code of statcheck, and all analyses can be found in the Supplementary Materials on OSF. Grades are considered to be sensitive data, and to avoid potential risks of privacy violation, these cannot be shared, in line with the General Data Protection Regulation (GDPR), which is a regulation in EU law on data protection and privacy (2016). After describing general characteristics of the coded master’s theses, we present Pearson correlations and point biserial correlations between pairs of thesis characteristics and between thesis characteristics and thesis grade. To correct for multiple testing, we use a rather stringent value of α ≤ .005. Table 2 lists descriptive statistics of thesis characteristics.

### 3.1. Thesis Characteristics

The mean grade of theses was 7.26; the lowest grade was 6.0 (minimum requirement for passing the exam), and the highest grade was 9.0. In total, 18.7% of theses received a grade ≥ 8.0 (*n* = 56), and are, depending on their other course grades, eligible for a *cum laude* distinction. The mean thesis length was 23.19 pages (min: 10, max: 54). The mean number of additional pages was 10.83 (min: 5, max: 85), and 39% of the theses had more than 10 additional pages. Most theses were not preregistered; 11 theses were preregistered (3.7%), and 6 (2.0%) included an URL to their own preregistration. Most theses explicitly listed their hypotheses (*n* = 207, 69%). These theses listed on average 4.02 hypotheses (min: 1, max: 15), with 75.8% of these theses (*n* = 157) listing either 2, 3, or 4 hypotheses. Furthermore, most of the theses investigated a moderation or interaction effect (*n* = 175, 58.3%), mediation analyses were less frequent (*n* = 95, 31.7%), 9.3% of the theses (*n* = 28) contained both types of analyses, and 19.3% contained neither (*n* = 58). Most theses described one study (*n* = 289, 96.3%); some described two (*n* = 9, 3%) or three (*n* = 2, 0.7%) studies. The mean sample size in a thesis was 1280.3; however, this result was skewed due to some studies with extremely large sample sizes (min: 6, max: 487,679). The 25th, 50th (median), and 75th percentiles of sample size were 135, 214, and 338, respectively. With these sample sizes, 9.7% (*n* = 29), 77.3% (*n* = 232), and 95.7% (*n* = 287) of the theses would have had at least 80% power to detect a small, medium, or large true effect in a two-tailed independent-sample *t*-test, respectively. In total, 47% (*n* = 141) of the theses conducted some form of power analysis, where most theses (39%, *n* = 117) reported an a priori power analysis, some reported post hoc power analysis (7.7%, *n* = 23), and only one thesis (0.3%) reported both.

Two theses did not describe any a priori hypotheses (0.7%). Of the remaining 298 theses, we coded the focal hypothesis based on the title in 91.6% of theses (*n* = 273); 41.3% from the title only (*n* = 123); 39.6% from the title combined with the first related hypothesis in the abstract (*n* = 118); 6.4% from the first hypothesis mentioned in the text that was related to the title (*n* = 19); and 4.4% from the title combined with prioritization of one hypothesis in the text (*n* = 13). For the remaining theses, the focal hypothesis was either based on the first hypothesis mentioned in the abstract (6.7%, *n* = 20), based on the first hypothesis in the text (0.7%, *n* = 2), based on prioritization of one hypothesis in the text (0.3%, *n* = 1), or based on other reasons noted by the coders (0.7%, *n* = 2). In total, 31.9% of focal hypotheses contained only a moderation effect (*n* = 95), 20.5% contained only a mediation effect (*n* = 61), 1.3% of focal hypotheses described both (*n* = 4), and 46.3% (*n* = 138) of focal hypotheses contained neither.

The mean number of focal results reported in a thesis was 4.63 (min: 0, max: 159, median: 2). However, 3.7% (*n* = 11) of studies did not report any statistical results concerning their focal hypotheses, nearly half of theses contained only 1 or 2 focal results (47.3%, *n* = 141), and 7.4% of theses (*n* = 22) reported 10 or more results. In 24.7% of theses, all focal results were statistically significant (71 out of 287 theses with focal hypothesis results), whereas 43.2% of theses (*n* = 124) presented only non-significant results. In 32.1% of theses (*n* = 92), the results were mixed, that is, some of the results were statistically significant and others were not. The total number of results reported in all theses was 1380, and 44.8% of these results (618) were statistically significant. In 117 theses (39.3%), an effect size was reported for the focal hypothesis.

With respect to the decision made regarding the focal hypothesis, half of the theses (*n* = 149) did not reject the null hypothesis of their focal hypothesis, whereas the other half (*n* = 147) concluded that there was at least some effect in some direction. Two theses did not draw a conclusion regarding the focal hypothesis. When results were mixed (92 theses), students concluded there was at least some effect in 70.7% of theses (*n* = 65), and they concluded that there was no effect in 28.3% of theses (*n* = 26).

Almost one in four theses (24.3%) provided some sort of distinction between confirmatory versus exploratory hypotheses or analyses, almost half of the theses discussed the assumptions of statistical tests (49.7%), and 41.7% of students provided at least one confidence interval within their thesis.

Statcheck found results that matched the APA format in 229 of the 300 theses. Although manual inspection showed that all theses contained frequentist statistics, statcheck could not automatically extract any statistics in 71 theses (23.7%). Within the remaining 229 theses, statcheck detected a total of 2052 results, with 8.96 results per thesis on average (*sd* = 8.9, min: 1, max: 77). Of these 2052 results, 276 were errors (13.5%), and 50 of those errors were gross inconsistencies (2.4%). Furthermore, a statistical reporting error was detected in 34.7% of all theses (*n* = 104), and 11.3% of the theses (*n* = 34) contained at least one gross inconsistency. In most of the theses where statcheck found results, it did not find any errors (*n* = 125 out of 229, 54.6%), or one or two errors (*n* = 69 out of 229, 30.1%). The maximum number of errors in one thesis was 11 errors. Gross inconsistencies occurred only once in 26 theses (11.4%), and 8 theses (3.5%) had multiple gross inconsistencies, with a maximum of seven.

### 3.2. Associations Between Thesis Characteristics and Their Associations with Thesis Grade

Table 3 shows the Pearson correlations between the thesis variables; α < .005 was used to assess the significance of the associations. All variables were either continuous (e.g., number of pages, number of results), or dichotomous (e.g., preregistered, decision). Correlations were large (i.e., about 0.5 or higher), between variables that are naturally positively associated, such as the number of results for the focal hypothesis and the number of statistically significant results (*r* = 0.95), the presence of any complex hypothesis and the presence of a focal hypothesis (*r* = 0.51), and the number of reporting errors and the number of gross inconsistencies (*r* = 0.46). Furthermore, large correlations were found between thesis length and appendix length (*r* = 0.47), number of hypotheses and thesis length (*r* = 0.52), and number of hypotheses and appendix length (*r* = 0.44). Thesis and appendix length were also moderately correlated (i.e., about 0.3) with making an explicit distinction between confirmatory and exploratory hypotheses or analyses (*r* = 0.30 and *r* = 0.31, respectively).

The decision of having at least some effect in some direction correlated with the number of statistically significant results for the focal hypothesis (*r* = 0.29), and it correlated negatively with having a complex focal hypothesis (*r* = −0.20). Providing many results for the focal hypothesis (an indicator of the QRP multiple testing) did not result in finding an effect more often (*r* = 0.15). Surprisingly, sample size was neither related to conducting a power analysis (*r* = 0.06), nor to the number of statistically significant results (*r* = 0.07).

Some of the RRP indicators correlated positively, whereas others showed a negative correlation with each other. Conducting a power analysis was positively correlated with providing an effect size for the focal hypothesis (*r* = 0.17); effect sizes for focal hypotheses and assumption tests often co-occurred (*r* = 0.17); and confidence intervals were provided more often when a complex hypothesis was investigated in the thesis (*r* = 0.24), and when the focal hypothesis was complex (*r* = 0.20). However, preregistration was not associated with providing a distinction between confirmatory and exploratory hypotheses or analyses (*r* = 0.14), and assumption tests occurred less often when a distinction between confirmatory and exploratory analyses had been made (*r* = −0.24). All results suggest that adopting some responsible research practices does not necessarily mean that all other responsible research practices are adopted as well.

The presence of reporting errors was positively correlated with thesis length (*r* = 0.20) but was not associated with having (and testing) more hypotheses (*r* = 0.18). Surprisingly, the presence of reporting errors was also positively correlated with reporting a power analysis (*r* = 0.18) and providing a distinction between exploratory and confirmatory analyses (*r* = 0.25). Reporting errors and gross inconsistencies showed no relation to the number of significant results for the focal hypothesis or the decision regarding the presence of an effect. Hence, here too, questionable research practices are hardly, if at all, correlated in master’s theses.

Table 4 reports all correlations of coded thesis characteristics and Statcheck results with thesis grade. Using α = .005 to adjust for multiple testing, only thesis length was associated with thesis grade, with a small-to-medium effect size (*r* = 0.188). Observed correlations of grade with other coded thesis characteristics ranged from *r* = −0.104 to *r* = 0.136. A multiple regression showed that all 18 predictors together did not explain thesis grade (*R^2^* = .094, adj. *R*^2^ = .036, *F*_(18,279)_ = 1.62, *p* = .056). Additionally, none of the types of thesis characteristics contributed to the explanation of thesis grade after controlling for the effect of predictors of other types: RRP (*F*_(7,279)_ = 0.93, *p* = .48), neutral (*F*_(3,279)_ = 2.29, *p* = .08), QRP (*F*_(3,279)_ = 2.12, *p* = .10), hypotheses (*F*_(5,279)_ = 1.02, *p* = .40). See Table 4 for the categorization of the predictors into the four types.

## 4. Discussion

Relating our findings to what we know about published manuscripts in the social sciences in general, master’s theses generally fared better on variables indicative of research quality than published manuscripts. Students explicitly listed their hypotheses in 69% of the theses, which is much higher than the prevalence of the word ‘research question’ in the main body of the text of papers in the social sciences (around 19%) and psychology and cognitive science (about 9%) (Thelwall & Mas-Bleda, 2020). Thelwall and Mas-Bleda (2020, p. 744) also report that hypotheses and research questions were stated much more frequently in U.S. doctoral dissertations than in research articles in several fields. The sample sizes theses reported were notably larger than sample sizes in published manuscripts, with quartiles of 135, 214, and 338, versus 33, 62, and 119 in published manuscripts in psychology (Hartgerink et al., 2017). This means that the statistical power of statistical tests in students’ master’s theses was higher than the power typically reported in published manuscripts, assuming that true effects, study designs, and significance levels do not differ (Fritz et al., 2013; Kühberger et al., 2014; Szucs & Ioannidis, 2017). Power analyses were reported in almost half of the theses, whereas this was estimated at between 3% and 5% in the published literature (Fritz et al., 2013; Kühberger et al., 2014), and at most one third amongst German psychology students (Krishna & Peter, 2018). Thorough reporting of statistics was also more prevalent among master’s theses. Confidence intervals were provided in 40.2% of theses, much more than the 10% estimated in published manuscripts. Furthermore, effect sizes were reported in 38% of published manuscripts, comparable to the prevalence of effect sizes reported for the focal hypothesis in theses (39%). However, for theses, this is a lower bound since effect sizes could also be reported in any of the other statistical results. Finally, the percentage of preregistered theses was comparable to the 3% of published psychology papers that Hardwicke et al. (2021) found.

All in all, our results show that theses provide more evidence of RRP than the published literature does. Perhaps students engage in RRP more than researchers, or researchers are not accustomed to reporting these details in a manuscript for publication. Articles are often restricted to a stricter maximum word count than theses, and perhaps the RRP indicators that students report are considered not relevant enough in the research report (by either the researcher, the reviewer, or the editor). However, this does not hold for differences in sample size; perhaps students focus more on single-study research, whereas researchers write multiple study papers, possibly encouraged by editors and current trends in psychology (Giner-Sorolla, 2012).

We also found that, next to statistical power, the number of statistically significant results was more balanced in theses than in the published literature. About 50% of theses concluded that there was some effect in some direction, which is much lower than 91.5% of published psychology and psychiatry papers that reported support for the tested hypothesis (Fanelli, 2010). Statistical power in theses is also higher than in published articles; if theses were investigating an effect of *d* = 0.25 with a median sample size of 214, and significance level of .05, in a two-tailed independent *t*-test, we would expect 44% of these tests to be statistically significant, as opposed to a power equal to 16% for published papers (median sample size of 62). Assuming that the true underlying effect sizes in psychology are indeed somewhere between small and medium (Gignac & Szodorai, 2016; Hartgerink et al., 2017; Open Science Collaboration, 2015), the observed number of statistically significant results (45%) seems to be in balance with the sample sizes used and statistical power in theses, suggesting no or less publication bias or selective reporting of statistical results.

Students’ master’s theses also provided better results than the published literature with respect to characteristics suggesting QRPs. Using statcheck, we identified reporting errors in almost 35% of theses, and 11% of all theses showed gross inconsistencies. Although these prevalences are higher than Olsen et al. (2019) found in theses, these results are slightly lower than the 50% and 12.9% of psychology articles that contained a reporting error or a gross inconsistency (Nuijten et al., 2016). The articles Nuijten et al. (2016) studied had a median number of 11 null hypothesis significance testing results per article, whereas our study found on average 8.96 results per article. This difference could partially explain the difference between our results and those by Nuijten et al. (2016). Olsen et al. only considered errors in the focal result, whereas we considered errors in all reported results, and this may explain the higher prevalences we found.

Counterintuitively, the use of one QRP or RRP was not or only weakly correlated to the use of any other QRP or RRP. Some RRPs even correlated negatively with each other, whereas some RRPs and QRPs were correlated positively. This indicates that RRPs do not necessarily co-occur, nor do they exclude QRPs. Hence, a unidimensional construct of “responsible scientific behavior” likely does not exist, going against the intuition of having both scientifically irresponsible scientists on the one hand and responsible ones on the other hand. We recommend examining the associations between the use of QRPs, or RRPs, in the published literature, as this association is currently unknown.

Master’s theses showed more evidence of RRPs than published manuscripts and less evidence of QRPs. The results of the Tilburg sample are comparable to the results of the earlier studies among student theses by Olsen et al. (2019) and Krishna and Peter (2018). Apparently, students were taught how to conduct research and how to write theses in a more responsible way than researchers behave when they write their own scientific manuscript. This is in line with research by O’Boyle et al. (2017), which showed that manuscripts were mainly distorted in the transformation from dissertation to published article. Master’s theses might even be subjected less to QRPs than dissertations, since supervision is closer and students might have little if anything to gain from conducting QRPs. The content of theses seems to be less subjected to biases and QRPs than the average published manuscript. This begs the question of whether our students conduct more responsible research than researchers. If researchers teach their students how to conduct research according to textbook rules, why do they forgo RRPs and engage in QRPs when publishing a manuscript? Many factors may contribute to the explanation for this, including different rules for publishing (e.g., word limits), evaluation (e.g., different emphasis on statistically significant results), performance pressure, etc. More research is needed into possible explanations for the difference in use of RRPs and QRPs in theses and published manuscripts. The thesis grade was (weakly) correlated with thesis length, but not with any of the other coded thesis variables related to RRPs and QRPs, including study sample size and the number of statistically significant focal results. This is surprising, since many of these RRP characteristics are typical of thorough, high-quality research, whereas indicators of QRPs are warning signals of false positives and overestimated effect sizes. Future research could focus on whether teachers are indifferent to RRPs and QRPs in student theses, whether they do not recognize these characteristics as indicators, or whether teachers simply do not find these characteristics relevant when determining grades. This could be investigated, for example, in an experimental setup, or by asking teachers about these characteristics explicitly. At Tilburg University, graders (the thesis supervisor plus a second assessor) are researchers themselves. It would therefore be interesting to see whether there is a relationship between the teachers’ own research behavior and published papers, and what the teacher values when grading theses, as well as the impact on the research behavior and output of the student. The attitude of the supervisor and the type of mentoring have shown to affect students’ attitude and behavior (Anderson et al., 2007; Gopalakrishna et al., 2022a, 2022b; Krishna & Peter, 2018). It seems therefore logical to assume that the same would uphold for their output.

A limitation of our study is that we studied psychology theses from one Dutch university, making generalization more difficult. Given that the Dutch master’s program duration is one year and mainly consists of courses focusing on the content of the specialization rather than research methodology, we expect that many of the basic research knowledge and skills are already acquired before starting the master’s program. Although some of the students in our sample have an international background, most students are likely to have followed a Dutch BSc program prior to their master’s program. Dutch BSc programs might have a larger focus on research methods compared to programs in other countries, such as the USA. The results of the current study, however, seem to be highly comparable with studies conducted at other universities and in other countries. A comparison of the curricula and final requirements could clarify differences between countries.

A second limitation is that a meaningful comparison between the five different master’s programs was hampered by too little statistical power. Furthermore, we also have no way of distinguishing the results of the published literature into different psychology research fields. Both researchers and publications cannot be placed in distinct categories. We chose not to make this distinction in comparisons in the current research project, and we do not know if they are comparable.

Thirdly, our results may be biased because of the theses excluded. Many theses were ‘confidential’, and in theory they could differ with respect to QRPs and RRPs from available theses. Moreover, all theses in our sample received a sufficient grade (at least 6 out of 10). Some of the included theses might have been the result of a resit, and it would be interesting to know if changes between the insufficient thesis version and the sufficient version are related to the use of QRPs and RRPs investigated here. Although the omission of (initially or finally) insufficient theses may have led to bias in our results, potential bias is likely small as the large majority of the theses are rated as sufficient.

A fourth limitation is that coding some of the variables proved difficult. Despite multiple rounds of double-coding and all the effort that was put into the coding manual, subjectivity could not be fully eliminated. In particular, the number of results pertaining to the focal hypothesis was difficult to code, which also impacted the coding of the number of statistically significant results. These numbers were difficult to code because theses seldom stipulate what the focal hypotheses are.

A fifth limitation concerns the interpretation of our findings when comparing them to published literature in the social sciences. Our comparisons include results of articles on student theses and on published manuscripts in different fields and different time-points further in the past. We realize that researcher and master’s student behavior differs across fields and may improve over the years. If comparing their manuscripts is the goal, then we recommend systematically comparing theses to published manuscripts in the same field and year(s).

Although this study aimed to be ecologically valid, we recommend conducting an experimental study. Such a study would be comparable to studies that investigated the impact of manuscript characteristics in peer review (e.g., Atkinson et al., 1982). In such a study, the use of RRPs and QRPs could be manipulated, and its effect on grading assessed.

## Figures and Tables

**Table 1:** Coded characteristics of all psychology master’s theses.

Category		Coded Information
General characteristics	1	Cohort year
	2	Master’s title
	3	Language (*‘Dutch’, ‘English’*)
	4	Thesis title
	5	Length of main text (number of pages)
	6	Length of references and supplementary materials (number of pages)
	7	Use of significance testing in thesis (*‘Yes’, ‘No’*)
	8	Number of studies in thesis
	9	Preregistered (*‘No’, ‘Yes, but no link’, ‘Yes, link available’*)
Design	10	Hypotheses explicitly listed (*‘Yes’, ‘No’*)
	11	If Yes: Number of hypotheses
	12	Moderation in any hypothesis/analysis (*‘Yes’, ‘No’*)
	13	Mediation in any hypothesis/analysis (*‘Yes’, ‘No’*)
	14	Sample size
	15	Power analysis (*‘No’, ‘A priori power analysis’, ‘Post hoc power analysis’, ‘Both a priori and post hoc power analysis’*)
Focal hypothesis	16	Text focal hypothesis
	17	Location of focal hypothesis (*‘Title’, ‘Title-text’, ‘Title-abstract’, ‘Title-first’, ‘Text’, ‘Abstract’, ‘First’, ‘Own description’*)
	18	Moderation in focal hypothesis *(‘Yes’, ‘No’*)
	19	Mediation in focal hypothesis *(‘Yes’, ‘No’*)
	20	Number of results on focal hypothesis
	21	Number of significant results of focal hypothesis
	22	Effect size for focal hypothesis (*‘ES available’, ‘ES missing’*)
	23	Decision on focal hypothesis (*‘Reject H0 (effect)’, ‘Partially reject H0 (some effect)’,‘Don’t reject H0 (no effect)’, ‘Marginal effect’, ‘Effect in opposite direction’, ‘Inconclusive (no decision)’*)
Reporting details	24	Distinction between exploratory–confirmatory hypotheses/analyses (*‘Yes’, ‘No’*)
	25	Assumption tests reported (*‘Yes’, ‘No’*)
	26	Confidence intervals given (*‘Yes’, ‘No’*)
Statcheck	27	Number of *p*-values detected
	28	Number of inconsistencies
	29	Number of gross inconsistencies

**Table 2:** Thesis characteristics, with categories general characteristics, design, focal hypothesis, reporting, and statcheck.

Variable	Percentage (Frequency) or Mean (SD)
General characteristics	*n* = 300
Grade	7.26 (0.67)
Language	
*‘Dutch’*	22.3% (67)
*‘English’*	77.7% (233)
Thesis length of main text (# pages)	23.19 (6.73)
Length of additional pages (# pages)	10.83 (8.26)
Preregistered	
*‘No’*	96.3% (289)
*‘Yes, but no link’*	1.7% (5)
*‘Yes, link available’*	2.0% (6)
Design	*n* = 300
Hypotheses explicitly listed	69% (207)
Number of hypotheses	4.02 (2.26)
Moderation in thesis	58.3% (175)
Mediation in thesis	31.7% (95)
Number of studies	1.04 (0.23)
Sample size per study	1280.3 (14,117.39)
Power analysis:	
*‘No’*	53% (159)
*‘A priori power analysis’*	39% (117)
*‘Post hoc power analysis’*	7.7% (23)
*‘Both a priori and post hoc power analysis’*	0.3% (1)
Focal hypothesis	*n* = 298
Moderation focal	33.2% (99)
Mediation focal	21.8% (65)
Number of focal results	4.63 (11.46)
Number of significant focal results	2.12 (5.90)
Effect size reported focal hypothesis	39.3% (181)
Focal hypothesis decision	
*‘Reject H0 (effect)’*	26.2% (78)
*‘Partially reject H0 (some effect)’*	15.4% (46)
*‘Don’t reject H0 (no effect)’*	50% (149)
*‘Marginal effect’*	1.3% (4)
*‘Effect in opposite direction’*	6.4% (19)
*‘Inconclusive (no decision)’*	0.7% (2)
Reporting	*n* = 300
Distinction exploratory–confirmatory	24.3% (73)
Assumption tests reported	49.7% (149)
Confidence intervals in thesis	41.7% (125)
Statcheck	*n* = 300
Total number of theses with inconsistencies	34.7% (104)
Total number of theses with gross inconsistencies	11.3% (34)
Mean number of errors per thesis	0.92 (1.95)
Mean number of gross inconsistencies per thesis	0.17 (0.62)

**Table 3:** Pearson correlations between coded thesis characteristics and statcheck results.

	1	2	3	4	5	6	7	8	9	10	11	12	13	14	15	16	17	18
Language (Engl)	--																	
2. Thesis length	−0.05	--																
3. Appendix length	0.12 *	0.47 **	--															
4. Preregistered	−0.07	0.06	−0.08	--														
5. # hypotheses	−0.04	0.47 **	0.37 **	−0.01	--													
6. Complex any hypothesis	0.12 *	0.01	0.05	−0.08	0.05	--												
7. Sample size	0.03	0.07	0.04	−0.01	0.03	0.03	--											
8. Power analysis	0.17 **	0.02	−0.01	0.07	0.14 *	0.04	0.06	--										
9. Complex focal hypothesis	−0.05	−0.09	−0.02	−0.03	−0.01	0.51 **	−0.07	0.01	--									
10. # results focal hypothesis	−0.22 **	0.21 **	0.20 **	−0.00	0.03	−0.03	0.04	−0.11	−0.11	--								
11. # sig results focal hypothesis	−0.18 **	0.21 **	0.22 **	−0.00	0.08	−0.02	0.07	−0.12 *	−0.16 *	0.95 **	--							
12. Effect size focal hypothesis	0.07	0.03	−0.01	0.02	−0.01	−0.05	−0.04	0.17 **	−0.08	−0.05	−0.04	--						
13. Decision focal hypothesis (any effect)	−0.05	0.04	−0.02	0.02	−0.03	−0.08	0.06	−0.04	−0.20 **	0.15 *	0.29 **	0.07	--					
14. Distinction confirmatory/exploratory	0.17 **	0.30 **	0.31 **	0.14 *	0.26 **	0.04	0.11	0.04	−0.09	−0.06	−0.03	0.01	0.02	--				
15. Assumption tests	−0.17 **	0.06	−0.09	−0.05	−0.06	0.11 *	−0.06	0.03	0.18 **	0.11	0.08	0.17 **	−0.10	−0.24 **	--			
16. Confidence intervals	0.05	0.12 *	0.10	−0.02	0.09	0.24 **	0.07	−0.01	0.20 **	−0.07	−0.06	−0.08	−0.03	−0.04	−0.06	--		
17. # reporting errors	0.06	0.20 **	0.16 *	−0.06	0.26 **	0.08	−0.03	0.18 **	−0.09	0.09	0.08	0.03	0.03	0.25 **	−0.10	−0.06	--	
18. # gross inconsistencies	0.00	0.05	0.03	−0.02	0.14 *	0.10	−0.01	0.06	−0.04	0.04	0.04	−0.07	0.06	0.07	−0.02	−0.05	0.46 **	--

Note. FH means focal hypothesis; * means *p* < .05, ** means *p* < .005.

**Table 4:** Pearson rank correlations between thesis grade and coded thesis characteristics (Neu[tral], Hyp[othesis], QRP, RRP).

	Correlation with Grade	*t*-Test	df	*p*-Value
Language (English)—Neu	0.087	1.514	298	.131
Thesis length—Neu	0.188 *	3.312	298	.001 *
Appendix length—Neu	0.135	2.358	298	.019 *
Preregistered—RRP	0.125	2.175	298	.030
# hypotheses—Hyp	0.073	1.261	298	.209
Complex any hypothesis—Hyp	0.098	1.691	298	.092
Sample size—RRP	0.021	0.356	298	.722
Power analysis—RRP	0.011	0.182	298	.856
Complex focal hypothesis—Hyp	−0.013	−0.228	296	.820
# results focal hypothesis = QRP	−0.035	−0.608	296	.544
# sig results focal hypothesis—Hyp	−0.011	−0.195	296	.846
Effect size focal hypothesis—RRP	−0.000	−0.006	296	.995
Decision focal hypothesis (any effect)—Hyp	−0.035	−0.597	296	.551
Distinction confirmatory/ exploratory—RRP	0.136	2.374	298	.018 *
Assumption tests—RRP	0.033	0.577	298	.565
Confidence intervals—RRP	0.066	1.145	298	.253
# reporting errors—QRP	−0.006	−0.104	298	.917
# gross inconsistencies—QRP	−0.104	−1.803	298	.072

Note: * *p* < 0.05.

## Data Availability

The original data presented in the study are openly available in the OSF page of this study (https://osf.io/b4g32/).

## Supplementary Materials

See the OSF page of this study (https://osf.io/b4g32/) for the supplementary materials, including the full coding protocol, data files, R-code, and interrater reliabilities.
